# The Clinical Impact of Continuing to Prescribe Antiretroviral Therapy in Patients with Advanced AIDS Who Manifest No Virologic or Immunologic Benefit

**DOI:** 10.1371/journal.pone.0078676

**Published:** 2013-11-15

**Authors:** David A. Wohl, Michelle A. Kendall, Judith Feinberg, Beverly Alston-Smith, Susan Owens, Suzette Chafey, Michael Marco, Sharon Maxwell, Constance Benson, Philip Keiser, Charles van der Horst, Mark A. Jacobson, for the A5030 Study Team

**Affiliations:** 1 University of North Carolina, Chapel Hill, North Carolina, United States of America; 2 Harvard School of Public Health, Boston, Massachusetts, United States of America; 3 University of Cincinnati, Cincinnati, Ohio, United States of America; 4 Division of AIDS, National Institutes of Health, Bethesda, Maryland, United States of America; 5 Frontier Science & Technology Foundation, Amherst, New York, United States of America; 6 University of California Los Angeles, Los Angeles, California, United States of America; 7 Columbia University, New York, New York, United States of America; 8 University of Washington, Seattle, Washington, United States of America; 9 University of California San Diego, San Diego, California, United States of America; 10 University of Texas – Southwestern Medical Center, Dallas, Texas, United States of America; 11 University of California San Francisco, San Francisco, California, United States of America; University of Pittsburgh, United States of America

## Abstract

**Introduction:**

Despite the efficacy and tolerability of modern antiretroviral therapy (ART), many patients with advanced AIDS prescribed these regimens do not achieve viral suppression or immune reconstitution as a result of poor adherence, drug resistance, or both. The clinical outcomes of continued ART prescription for such patients have not been well characterized.

**Methods:**

We examined the causes and predictors of all-cause mortality, AIDS-defining conditions, and serious non-AIDS-defining events among a cohort of participants in a clinical trial of pre-emptive therapy for CMV disease. We focused on participants who, despite ART had failed to achieve virologic suppression and substantive immune reconstitution.

**Results:**

233 ART-receiving participants entered with a median baseline CD4+ T cell count of 30/mm^3^ and plasma HIV RNA of 5 log_10_ copies/mL. During a median 96 weeks of follow-up, 24.0% died (a mortality rate of 10.7/100 patient-years); 27.5% reported a new AIDS-defining condition, and 22.3% a new serious non-AIDS event. Of the deaths, 42.8% were due to an AIDS-defining condition, 44.6% were due to a non-AIDS-defining condition, and 12.5% were of unknown etiology. Decreased risk of mortality was associated with baseline CD4+ T cell count ≥25/mm^3^ and lower baseline HIV RNA.

**Conclusions:**

Among patients with advanced AIDS prescribed modern ART who achieve neither virologic suppression nor immune reconstitution, crude mortality percentages appear to be lower than reported in cohorts of patients studied a decade earlier. Also, in contrast to the era before modern ART became available, nearly half of the deaths in our modern-era study were caused by serious non-AIDS-defining events. Even among the most advanced AIDS patients who were not obtaining apparent immunologic and virologic benefit from ART, continued prescription of these medications appears to alter the natural history of AIDS—improving survival and shifting the causes of death from AIDS- to non-AIDS-defining conditions.

## Introduction

Potent antiretroviral therapies have drastically improved the disease-free survival of HIV-infected patients and have provided millions of years of additional life to those receiving these drugs [1], [2]. However, the benefits of such therapies have not been universal and HIV-infected individuals in resource-rich regions continue to die prematurely [3], [4], particularly those who fail to achieve immune reconstitution from antiretroviral therapy [5].

Nevertheless, even among patients with advanced AIDS (i.e., absolute CD4+ T cell count <50/mm^3^) who fail to achieve any substantive immune reconstitution, suppression of HIV replication with ART results in survival rates that far exceed the optimal rates reported before the modern ART era. For example, in the COHERE study of 75,336 HIV+ patients observed between 1997 and 2010, the mortality rate among patients with a most recent absolute CD4+ T cell count <50/mm^3^ was only 6.5% per year of suppressed viral load [6]. In contrast, in a number of randomized trials conducted in the early 1990s that were designed to test combination nucleoside reverse transcriptase inhibitor (NRTI) drug combinations or prophylaxis to prevent *M. avium complex* infection, crude mortality percentages of 32%–40% over one to two years of follow-up were reported [7]–[9].

However, it is not clear what clinical impact continuing to prescribe ART for late-stage AIDS patients who demonstrate *neither* suppression of HIV replication *nor* substantive improvement in absolute CD4+T cell count has in the modern ART era. Results of a pivotal, randomized, strategic treatment interruption study conducted in the modern ART era suggested that continuing ART in AIDS patients with multi-drug-resistant HIV and unsuppressed viremia has clinical efficacy compared to temporarily stopping treatment, although not with respect to survival [10]. However, the degree of medication adherence in this trial was very high, and patients randomized to continue failing ART had some degree of immune reconstitution during follow-up (an average increase in the median absolute CD4+ T cell count by approximately 50 cells/mm^3^). There are also little data regarding the cause of death in patients with advanced AIDS who fail to achieve either virologic suppression or substantive immune reconstitution with continued ART. Whether the causes of death among these individuals mirror those of the pre-modern ART era or are influenced by receipt of ART is not clear.

To address these questions, we examined mortality and its causes among a cohort of participants in a trial of pre-emptive therapy for cytomegalovirus viremia. The cohort consisted of patients who were receiving a combination of at least three antiretroviral agents for at least three months, had failed to suppress HIV replication (plasma HIV RNA level greater than 1,000 copies/mL), and had not achieved an absolute CD4+ T cell count above 100 cells/mm^3^ prior to enrollment.

## Methods

### Participants

The Biomedical Institutional Review Board at the University of North Carolina and the institutional review boards at each participating AIDS Clinical Trials Group study sites approved the protocol and all participants provided verbal and written informed consent. AIDS Clinical Trials Group (ACTG) study A5030 was a prospective, double-blind, placebo-controlled, multi-centered, randomized trial conducted at academic centers across the US between 2000 and 2005. Participants were: CMV seropositive (IgG); without evidence of CMV end-organ disease; CD4+ T cell count <100 cells/mm^3^; and plasma HIV RNA >400 copies/mL within 30 days prior to entry. Participants must either have been receiving antiretroviral therapy (ART) continuously for at least three months, or not be receiving and not planning to initiate ART. ART was defined as at least a three-drug antiretroviral regimen, including at least one protease inhibitor (PI) and/or non-nucleoside reverse transcriptase inhibitor (NNRTI). Participants on a three-drug nucleoside reverse transcriptase inhibitor (NRTI) regimen were eligible if treated in the past with a PI or an NNRTI. ART was prescribed as part of usual clinical care and not determined by the study protocol. The HIV treatment criteria were designed to lead to the enrollment of patients who, for various reasons such as viral resistance and/or medication non-adherence, were not receiving substantive benefit from ART and thus were at risk of CMV end-organ disease.

The study design has been described previously and included enrollment of participants into an observation phase during which plasma CMV DNA PCR was collected at regular intervals [11]. Those with detectable CMV entered into a randomization phase and were assigned valganciclovir or placebo. Because there were no differences between arms in the survival and CMV end organ disease rates during the randomization phase, morbidity and mortality data from all participants who were receiving ART for at least three months and had a suboptimal virologic and immunologic response to ART were included in the present analysis. We defined suboptimal ART response as an absolute CD4+ T cell count below 100 cells/mm^3^ and a plasma HIV RNA level greater than 1,000 copies/mL at enrollment. This viral load threshold was selected to exclude those with chronic low-level viremia and virologic ‘blips’. Of the 338 participants in the trial, 262 (77.5%) entered the study on ART and, of these, 233 (88.9%) met these criteria for suboptimal ART response.

### Evaluations

Clinical evaluations were conducted every 4–12 weeks. Lymphocyte subsets were obtained at baseline and every 16 weeks. Quantitative plasma HIV RNA PCR (standard HIV-1 RT PCR, Roche Diagnostics. Indianapolis, IN: lower limit of detection of 400 copies/mL, respectively) was obtained at baseline.

### Categorization of Cause of Death

For participants who died during study follow-up, the causes of death reported by the site investigators (primary cause, four contributing causes, and a descriptive narrative) were reviewed independently by the two of the study chairs (DAW and MAJ), who categorized each death as being due to a condition that was AIDS-defining (1993 Centers for Diseases Control and Prevention), non-AIDS defining, or of uncertain etiology. In the two cases where the categorization of a death by the two study chairs did not agree, an experienced HIV clinician (CvdH) not directly associated with the study reviewed the data and provided the final categorization.

### Statistical Methods

The primary endpoints were times to death, AIDS-defining condition, and serious non-AIDS event. In this study population participants were often diagnosed with both AIDS-defining conditions and serious non-AIDS events before dying or withdrawing from study. Thus, there were events other than the endpoint of interest that altered the probability of the endpoint. These events are competing risks.

For the time to death analysis, withdrawing from study was the competing risk. Time to death was calculated from study entry to date of death. For participants who withdrew from study before dying, event times were calculated from study entry to the date of last clinic visit.

For the time to new AIDS-defining condition analysis, dying and withdrawing from study were the competing risks. However, those dying from an AIDS-defining condition were endpoints in this analysis. Time to new AIDS-defining condition was calculated from study entry to date of diagnosis of the new AIDS-defining condition. For participants who died or withdrew from study before diagnosis of a new AIDS-defining condition, event times were calculated from study entry to the date of death or last clinic visit, respectively.

For the time to new serious non-AIDS event analysis, dying from an AIDS-defining condition or unknown cause of death and withdrawing from study were the competing risks. However, those who died from a new serious non-AIDS event were endpoints in this analysis. Time to new serious non-AIDS event was calculated from study entry to date of diagnosis of the new serious non-AIDS event. For participants who died or withdrew from study before diagnosis of a new serious non-AIDS event, event times were calculated from study entry to the date of death or last clinic visit, respectively.

In these time to endpoint analyses, participants who completed follow-up without an endpoint or competing risk event were censored, with times calculated from study entry to date of last clinic visit. Nonparametric cumulative incidence curves were used to estimate the cumulative incidence function of each endpoint of interest by accounting for the presence of competing risks. The cumulative incidence curves were fit using the cuminc function in S-PLUS (Version 3.4 Release 1 for Sun SPARC, SunOS 5.3 : 1996).

The associations between baseline variables and each time to endpoint of interest were examined using proportional hazards regression modeling of sub-distribution functions in competing risks [12]. Age, race, Karnofsky Performance Score (KPS), and CD4+ T cell count were *a priori* dichotomized based on commonly accepted practice (age, race) or clinical significance (CD4+ T cell count, KPS). Variables found to be statistically significant (p<0.10) in the univariable models were examined together in a multivariable model. If both the continuous and dichotomous versions of the variable were statistically significant in the univariable model, then the variable that was most significant was included in the multivariable model. The multivariable model was reduced using the backward elimination method until all variables were statistically significant at the 0.05-level. The competing risks proportional hazards regression models were fit using the CMPRSK package in R (Copyright 2010, The R Foundation for Statistical Computing Version 2.12.1 (2010-12-16), ISBN 3-900051-07-0).

Incidence rates were calculated using standard epidemiological methods with a 48-week year. Confidence intervals for incidence rates and crude mortality rates were calculated using the Poisson distribution and Wilson's binomial method, respectively, and were fit using the Stata command cii (Copyright 2009, StataCorp LP Version 11.1). Comparisons between groups were made using the Wilcoxon and sign tests for unpaired and paired continuous data, respectively.

## Results

### Participant Characteristics

From 2000 to 2004, 233 participants enrolled in the A5030 trial had been receiving ART prescriptions from a health care provider for at least three months prior to entry and were neither virologically suppressed below 1,000 copies/mL nor had substantive immunologic reconstitution at trial entry. The characteristics of this cohort are detailed in Table 1. The cohort was mostly male and racially diverse, with a median age of 42 years (range: 20–63). Their median baseline Karnofsky performance score (KPS) was 90 (range: 60–100), median nadir CD4+ T cell count prior to study entry (available for 211 participants) was 12/mm^3^ (range: 0–100), and median baseline CD4+ T cell count was 30/mm^3^ (range: 0–95). Their median baseline plasma HIV RNA was 5 log_10_ copies/mL (range: 3.04–6.48). There was minimal change in the antiretrovirals prescribed by participants' health care providers during study follow-up. The mean change in CD4+ T cell count among the 201 (86.3%) participants with at least one post-baseline value was 33 cells/mm^3^ (95% CI = (19, 48)), a small but statistically significant increase (sign test p = 0.027). On-study HIV RNA data were not collected.

**Table 1:** Baseline Characteristics.

Characteristic	Total(N = 233)
Male [N (%)]	209 (90%)
White, Non-Hispanic [N (%)]	131 (56%)
Black, Non-Hispanic [N (%)]	66 (28%)
Hispanic [N (%)]	32 (14%)
Age [years, median (range)]	42 (20–63)
Karnofsky Performance Score	
median (range)	90 (60–100)
<90 [N (%)]	102 (44%)
<80 [N (%)]	33 (14%)
Nadir CD4+ T Cell Count	
median (range) [cells/mm^3^]*	12 (0–100)
<25 [cells/mm^3^, N (%)]*	197 (70%)
<50 [cells/mm^3^, N (%)]*	253 (90%)
CD4+ T Cell Count	
median (range) [cells/mm^3^]	30 (0–95)
<25 [cells/mm^3^, N (%)]	99 (42%)
<50 [cells/mm^3^, N (%)]	175 (75%)
HIV RNA [log_10_ copies/mL, median (range)]	5.00 (3.04–6.48)

* Based on 211 participants with available nadir CD4+ T cell counts.

### Mortality

During a median follow-up of 96 weeks (mean: 108; range: 4–249), 56 (24.0% [95% CI = (19.0%–29.9%)]) of these 233 participants died, for a death incidence rate of 10.7/100 patient-years (95% CI = (8.1–13.9)). Of these, 42.8% (n = 24) were reportedly due to an AIDS-defining condition (1993 Centers for Diseases Control and Prevention) or AIDS progression, 44.6% (n = 25) were reportedly due to a non-AIDS-defining condition (non-AIDS-defining infectious or malignant diseases, pulmonary or cardiovascular conditions, or other diseases listed in Table 2), and 12.5% (n = 7) were of unknown etiology. The median baseline CD4+ T cell count of those who died was 13/mm^3^ (range: 0–84). At the visit prior to death, the median CD4+ T cell count was 7/mm^3^ (range: 0–135).

**Table 2:** Reported death causes, AIDS-defining conditions, and serious non-AIDS-defining events.

Death Causes (N = 56)	N (%*)	AIDS-Defining Conditions (N = 59)	N (%*)	Serious Non-AIDS Events (N = 40)	N (%*)
Progression of AIDS**	14 (25%)	Pneumocystis *jirovecii* pneumonia	11 (18%)	Sepsis	19 (48%)
AIDS-Defining Conditions***	10 (18%)	Cytomegalovirus	10 (17%)	Pulmonary Diseases	16 (40%)
Pneumocystis *jirovecii* pneumonia	3	Candidiasis	9 (15%)	Cardiovascular Diseases	1 (3%)
Wasting Syndrome	2	Cryptococcosis	6 (10%)	Skin Cancer	1 (3%)
Kaposi's Sarcoma	2	Wasting Syndrome	4 (6%)	Nephropathy	1 (3%)
CNS Lymphoma	2	Kaposi's Sarcoma	3 (5%)	CNS Disease	1 (3%)
Cytomegalovirus	1	Lymphoma	3 (5%)	Cirrhosis	1 (3%)
Sepsis	9 (16%)	PML****	3 (5%)		
Pulmonary Diseases	5 (9%)	Cryptosporidiosis	3 (5%)		
Cardiovascular Diseases	4 (7%)	CNS Lymphoma	2 (3%)		
Other Malignancies (Lung, Laryngeal)	2 (4%)	Zoster	1 (2%)		
Renal Failure	1 (2%)	Toxoplasmosis	1 (2%)		
Pulmonary Embolus	1 (2%)	Mycobacterium Avian Complex	1 (2%)		
Pancytopenia	1 (2%)	Isosporiasis	1 (2%)		
Lupus	1 (2%)	Coccidioidal Meningitis	1 (2%)		
Liver Failure	1 (2%)				
Unknown Etiology	7 (12%)				

* Total % does not add to 100% due to rounding.

** Reported death due to AIDS without AIDS-defining condition.

*** Centers for Disease Control and Prevention 1993 AIDS-Defining Conditions.

**** Progressive Multifocal Leukoencephalopathy.

**Figure 1:**
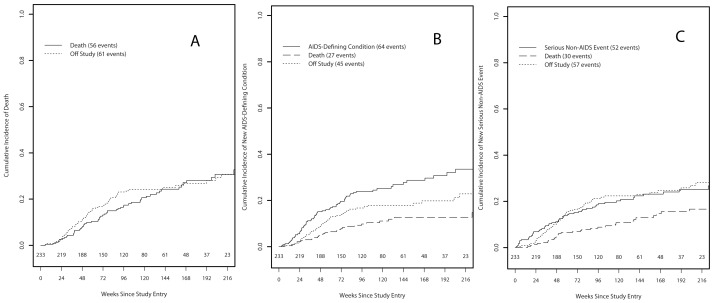
Death (56 events), Off Study (61 events) in panel [A]. [B] AIDS-Defining Condition (64 events), Death (27 events), Off Study (45 events). [C] Serious Non-AIDS Event (52 events), Death (30 events), Off Study (57 events).

The cumulative incidence curve of time to death is shown in Figure 1A. Withdrawal from study (n = 61) was the competing risk for dying. The reasons for study withdrawal included severe debilitation, unable to get to clinic, withdrawal of consent, and unable to contact. Participants who withdrew from study were not significantly different from those who died or were censored with respect to sex, race, age at entry, KPS, and baseline HIV RNA (p>0.10; data not shown). However, those who withdrew from study had significantly higher baseline CD4+ T cell counts than those who died, with a median of 32/mm^3^ (range: 2–83; Wilcoxon test p<0.001). At the visit prior to withdrawal, the median CD4+ T cell count was 23/mm^3^ (range: 0–386).

In univariable competing risks proportional hazards regression analyses, a decreased hazard of dying was significantly associated with baseline KPS ≥90 (HR = 0.57; 95% CI = (0.34,0.96); p = 0.036), higher nadir CD4+ T cell count (HR = 0.74 per 10-cells/mm^3^; 95% CI = (0.61,0.89); p = 0.002), nadir CD4 ≥25/mm^3^ (HR = 0.38; 95% CI = (0.18,0.81); p = 0.012), baseline CD4+ T cell count (HR = 0.75 per 10-cells/mm^3^; 95% CI = (0.64,0.87); p = 0.0002), baseline CD4 ≥25/mm^3^ (HR = 0.29; 95% CI = (0.16,0.52); p<0.0001), and baseline CD4 ≥50/mm^3^ (HR = 0.25; 95% CI = (0.10,0.63); p = 0.003; Table 3). In the final multivariable analysis, baseline KPS ≥90 (HR = 0.56; 95% CI = (0.32,0.97); p = 0.038) and nadir CD4+ T cell count (HR = 0.75 per 10-cells/mm^3^; 95% CI = (0.62,0.91); p = 0.003; Table 4) were significantly associated with a decreased adjusted hazard of dying.

**Table 3:** Univariable competing risks analyses of times to death, new AIDS-defining conditions, and new serious non-AIDS-defining events.

	Death			AIDS-Defining Conditions			Serious Non-AIDS Events		
Variable	Estimated HR	95% CI	P-Value	Estimated HR	95% CI	P-Value	Estimated HR	95% CI	P-Value
Baseline Age									
per 10-years older	1.11	(0.72, 1.69)	0.64	1.17	(0.81, 1.68)	0.41	0.94	(0.57, 1.55)	0.81
≥40 vs <40	0.91	(0.53, 1.57)	0.74	1.29	(0.76, 2.18)	0.34	1.06	(0.60, 1.88)	0.85
Sex									
male vs female	2.00	(0.63, 6.32)	0.24	1.73	(0.63, 4.76)	0.29	1.30	(0.45, 3.78)	0.62
Race									
white vs non-white	1.02	(0.603, 1.72)	0.95	1.49	(0.89, 2.50)	0.13	0.98	(0.56, 1.69)	0.93
black vs non-black	0.84	(0.46, 1.56)	0.59	0.27	(0.12, 0.61)	**0.002**	0.95	(0.51, 1.75)	0.87
Baseline Karnofsky Performance Score									
≥80 vs <80	0.60	(0.30, 1.23)	0.16	0.48	(0.25, 0.91)	**0.023**	0.83	(0.39, 1.77)	0.64
≥90 vs <90	0.57	(0.34, 0.96)	**0.036**	0.62	(0.38, 1.004)	**0.052**	0.81	(0.47, 1.39)	0.44
Nadir CD4+ T Cell Count									
per 10-cells/mm^3^ higher	0.74	(0.61, 0.89)	**0.002**	1.06	(0.95, 1.18)	0.32	0.85	(0.71, 1.03)	**0.094**
≥25 vs <25 cells/mm^3^	0.38	(0.18, 0.81)	**0.012**	1.33	(0.78, 2.25)	0.29	0.70	(0.37, 1.32)	0.27
≥50 vs <50 cells/mm^3^	(-)	(-)	DNC*	0.98	(0.45, 2.15)	0.96	0.53	(0.17, 1.64)	0.27
Baseline CD4+ T Cell Count									
per 10-cells/mm^3^ higher	0.75	(0.64, 0.87)	**0.0002**	0.94	(0.85, 1.04)	0.22	0.86	(0.75, 0.99)	**0.030**
≥25 vs <25 cells/mm^3^	0.29	(0.16, 0.52)	**<0.0001**	0.69	(0.42, 1.12)	0.13	0.47	(0.27, 0.82)	**0.007**
≥50 vs <50 cells/mm^3^	0.25	(0.10, 0.63)	**0.003**	0.74	(0.42, 1.28)	0.28	0.67	(0.34, 1.31)	0.24
Baseline HIV RNA									
per 1-log_10_ copies/mL lower	0.76	(0.49, 1.18)	0.22	0.60	(0.42, 0.87)	**0.007**	0.79	(0.52, 1.19)	0.26

* DNC = model did not converge.

**Table 4:** Multivariable competing risks analyses of times to death, new AIDS-defining conditions, and new serious non-AIDS-defining events.

	Death			AIDS-Defining Conditions			Serious Non-AIDS Events		
Variable	Estimated HR	95% CI	P-Value	Estimated HR	95% CI	P-Value	Estimated HR	95% CI	P-Value
Race									
black vs non-black	(-)	(-)	(-)	0.30	(0.13, 0.69)	**0.005**	(-)	(-)	(-)
Baseline Karnofsky Performance Score									
≥80 vs <80	(-)	(-)	(-)	0.50	(0.27, 0.94)	**0.030**	(-)	(-)	(-)
≥90 vs <90	0.56	(0.32, 0.97)	**0.038**	(-)	(-)	(-)	(-)	(-)	(-)
Nadir CD4+ T Cell Count; per 10-cells/mm^3^ higher	0.75	(0.62, 0.91)	**0.003**	(-)	(-)	(-)	0.97	(0.79, 1.20)	0.80
Baseline CD4+ T Cell Count									
≥25 vs <25 cells/mm^3^	(-)	(-)	(-)	(-)	(-)	(-)	0.46	(0.22, 0.94)	**0.034**
Baseline HIV RNA									
per 1-log_10_ copies/mL lower	(-)	(-)	(-)	0.66	(0.45, 0.96)	**0.031**	(-)	(-)	(-)

### AIDS-Defining Conditions

At least one new AIDS-defining condition developed in 25.3% (n = 59) participants receiving ART prescriptions at entry, with a median follow-up of 78 weeks (mean: 93; range: 3–249). Five additional participants died with an AIDS-defining condition, for a new AIDS-defining condition incidence rate of 14.2/100 person-years (95% CI = (10.9,18.1)). Pneumocystis *jirovecii* pneumonia, CMV (retinitis or colitis), esophageal candidasis, cryptococcal infection, wasting syndrome, KS, lymphomas, PML, and cryptosporidia infection together accounted for over 90% of these events (Table 2). The median CD4+ T cell count of those who developed a new AIDS-defining condition was 25/mm^3^ (range: 1–90) at entry and 13/mm^3^ (range: 0–127) at the visit before the new AIDS-defining condition diagnosis.

The cumulative incidence curve of time to new AIDS-defining condition is shown in Figure 1B. Death (n = 27) and withdrawal from study (n = 45) were competing risks for developing a new AIDS-defining condition. In univariable proportional hazards competing risks regression analyses, a decreased hazard of developing a new AIDS-defining condition was significantly associated with black race (HR = 0.27; 95% CI = (0.12,0.61); p = 0.002) baseline KPS ≥80 (HR = 0.48; 95% CI = (0.25,0.91); p = 0.023), baseline KPS ≥90 (HR = 0.62; 95% CI = (0.38,1.004); p = 0.052), and lower baseline log_10_ HIV RNA (HR = 0.60 per 1 log_10_ copies/mL; 95% CI = (0.42,0.87); p = 0.007; Table 3). In multivariable analysis, black race (HR = 0.30; 95% CI = (0.13,0.69); p = 0.005), baseline KPS ≥80 (HR = 0.50; 95% CI = (0.27,0.94); p = 0.030), and lower baseline HIV RNA (HR = 0.66 per 1-log_10_ copies/mL; 95% CI = (0.45,0.96); p = 0.031; Table 4) were significantly associated with a decreased adjusted hazard of developing a new AIDS-defining condition.

### Serious Non-AIDS-Defining Events

New serious non-AIDS-defining events were reported for 17.2% (n = 40) of the participants receiving ART prescriptions at entry; in an additional 12, the event was fatal. With a median follow-up of 81 weeks (mean: 98; range: 1–249), the new serious non-AIDS-defining event incidence rate was 11.0/100 person-years (95% CI = (8.2,14.4)). Sepsis and pulmonary diseases accounted for nearly 90% of these events (Table 2). The median CD4+ T cell count for those who reported a new serious non-AIDS-defining event was 15/mm^3^ (range: 0–89) at entry and 9/mm^3^ (range: 0–135) at the visit prior to the new serious non-AIDS-defining event.

The cumulative incidence curve of time to new serious non-AIDS-defining event is shown in Figure 1C. Death (n = 30) and withdrawal from study (n = 57) were competing risks for reporting a new serious non-AIDS-defining event. In univariable competing risks proportional hazards regression analyses, a decreased hazard of reporting a new serious non-AIDS event was significantly associated with higher nadir CD4+ T cell count (HR = 0.85 per 10-cells/mm^3^; 95% CI = (0.71,1.03); p = 0.094), higher baseline CD4+ T cell count (HR = 0.86 per 10-cells/mm^3^; 95% CI = (0.75,0.99); p = 0.030), and baseline CD4+ T cell count ≥25/mm^3^ (HR = 0.47; 95% CI = (0.27,0.82); p = 0.007); Table 3). With nadir CD4+ T cell count and baseline CD4+ T cell count in the multivariable model, only baseline CD4+ T cell count ≥25/mm^3^ remained significant (HR = 0.46; 95% CI = (0.22,0.94); p = 0.034; Table 4).

## Discussion

In a cohort of individuals with very advanced HIV disease who had been prescribed ART and failed to achieve complete virologic suppression and substantive immune reconstitution prior to enrollment between 2000 and 2004, mortality was lower than reported in comparable cohorts studied in the early 1990s (a time when modern antimicrobial prophylaxis for opportunistic infections and nucleoside-reverse-transcriptase-inhibitor [NRTI]-only antiretroviral regimens were widely available). The 233 participants in this cohort enrolled in the A5030 protocol between 2000 and 2004 with a median CD4+ T cell count of 30/mm^3^, plasma HIV RNA of 5 log_10_ copies/mL, and Karnofsky Performance Score of 90 had 24.0% (95% CI = (19.0%,29.9%) mortality during a median of 24.1 (mean: 26.9) months of follow-up. In contrast, 333 participants with a median CD4+ T cell count of 30/mm^3^ and mean Karnofsky Performance Score of 86 who were assigned to active drug in a randomized trial of clarithromycin for *M. avium complex* prophylaxis a decade earlier, had a 32% (95% CI = (27.3%,37.3%)) mortality during a mean 15 months of follow-up [7]. During this same time period, 699 participants with a median CD4+ T cell count of 23/mm^3^ who were assigned a variety of zidovudine and/or didanosine and/or zalcitabine antiretroviral regimens had 40% (95% CI = (36.5%,43.7%)) mortality after a median of 18 months of follow-up [8], and 566 participants with a CD4+ T cell count of 50–75/mm^3^ assigned to active drug in a randomized trial of rifabutin for *M. avium complex* prophylaxis had 35% (95% CI = (31.3%,39.2%)) mortality during a mean follow-up of approximately two years. This suggests that even in the most advanced AIDS patients who obtain little, if any, *objective* immunologic or virologic benefit from modern antiretroviral treatment, ART may improve survival compared to NRTI-only therapy.

One way by which ART might alter the natural history of AIDS in such “treatment failure” patients is to reduce the proportion of deaths caused by AIDS-defining conditions. In the HIV Outpatient Study, the proportions of deaths due to AIDS-defining conditions was 87% in 1996, prior to widespread use of modern ART regimens, but decreased to 57% by 2004 [13]. The rise of non-AIDS-defining conditions as a cause of death followed an incremental increase in CD4+ T cell counts at the time of death in this study (from 59/mm^3^ in 1996 to 287/mm^3^ in 2004), and non-AIDS deaths were associated with higher CD4+ T cell counts at the time of ART initiation. In our investigation, non-AIDS-defining causes of death equaled AIDS-defining causes of death despite much lower CD4+ T cell counts among those who died.

The finding of high rates of non-AIDS-defining causes of death in our cohort of patients with profoundly low CD4+ T cell counts mirrors data from a mortality analysis of the pooled antiretroviral-receiving control arms of the ESPRIT and SILCAAT trials, where rates of AIDS- and non-AIDS deaths were similar and low CD4+ T cell counts were associated with not only all-cause mortality, but also fatal non-AIDS events [14]. In that analysis, most of these deaths occurred among those with CD4+ T cell counts below 50/mm^3^, however, a substantial proportion was also observed at higher counts.

The shift over time in mortality from the AIDS events of the pre-modern-ART era to conditions that have not traditionally been considered a consequence of HIV disease progression suggests a potential protective effect of modern ART regimens in preventing death from AIDS-defining conditions, even among patients with persistent low CD4+ T cell counts and uncontrolled HIV replication. In the A5030 trial, when analyzing both the 272 antiretroviral-treated and the 65 -untreated participants, receipt of ART at study entry was significantly associated with a decreased hazard of developing a new AIDS-defining condition (HR = 0.44; 95% CI = (0.28,0.68); p = 0.0002, data not shown). That ART, even when not achieving suppression of HIV replication or substantive reconstitution of immune function, may provide some protection against opportunistic infections is further supported by our earlier finding of lower rates of CMV disease than expected based on pre-modern-ART era data in this cohort of patients with advanced AIDS [11].

Consistent with much larger cohort studies, 7% of the deaths among those included in this analysis were attributed to cardiovascular disease. Like several of the other non-AIDS-defining causes of death in our study, cardiovascular disease may be associated with chronic inflammation [15]. Markers of inflammation and coagulation were not collected; however, recent data point to a strong correlation between uncontrolled HIV replication and these markers [15]–[20]. It is possible that in the setting of prophylaxis against opportunistic infections and a partial protective ART effect, survival was long enough for conditions triggered or exacerbated by chronic inflammation to emerge.

Other than factors related to HIV therapy and CD4+ T cell count, there were few other covariates associated with serious and/or fatal outcomes. Blacks were less likely to develop a new AIDS-defining condition compared to non-blacks, but there were no racial differences in the risk of death or for serious non-AIDS-defining events.

There are limitations that should be considered when interpreting these findings. Foremost, the causes of death were those reported from ACTG study sites and were not determined through independent reviews of death certificates or primary, medical-record source documents. Based on the data that were available from the sites, categorization of the causes of death was made by the study chairs (both HIV clinicians) with an independent expert HIV clinician adjudicating two cases of discordant categorization. In addition, the cohort consisted of patients who were enrolled in a clinical trial, and the supervision afforded by the study may have led to greater than usual attention to prophylaxis of opportunistic infections and clinical monitoring. Lastly, many participants prematurely withdrew from follow-up, some due to debility that may have represented an AIDS-related or non-AIDS-related event. Therefore, the study outcomes competed with one another and with study withdrawal. However, the analysis methods accounted for these competing risks to appropriately summarize the survival curves and to appropriately assess associations between baseline covariates and the events of interest in the regression analyses. In addition, our comparisons to historical controls permit many biases. It is possible that virologically non-suppressive, immunologically ineffective ART is of no clinical benefit and that the difference in survival observed between the A5030 cohort and historical trials from a decade earlier represents other improvements in medical care for patients with advanced AIDS. Nevertheless, baseline CD4+ T counts of the three historical cohorts were comparable and follow-up was often shorter than in A5030 cohort. Further, the prophylaxis for opportunistic infections in advanced AIDS has changed little from the early 1990s to early 2000s (only the active *M. avium complex* prophylaxis arms from historical trials were included in our comparisons).

In conclusion, patients receiving modern ART regimens but who, as a consequence of antiretroviral drug resistance and/or suboptimal medication adherence, had persistent very low CD4+ T cell counts and failed to achieve complete virologic suppression were found to be at high risk of death, particularly from non-AIDS causes, though lower than that reported in patients with comparable CD4+ T cell counts receiving optimal opportunistic infection prophylaxis and antiretroviral therapy in the pre-modern-ART era. The lower than expected rates of opportunistic conditions observed in this cohort also suggest a potential protective effect of apparently ineffective ART against the development of such conditions. Overall, these findings support continued prescription of ART to patients with advanced AIDS who appear to obtain little, if any, virologic or immunologic benefit from their HIV therapy.
